# What a Mother-Expectant Should Know

**Published:** 1912-06-22

**Authors:** 


### MATERNITY AND MOTHERING.

(Inquiries and Correspondence are invited.)

### What a Mother-Expectant Should Know.

Of books for the instruction of wives and mothers a very large choice exists; and, since the subject is one which naturally appeals to a fair percentage of the whole population, the demand for and circulation of books of this sort is very considerable. In merit, in scope, in size, and in popularity wide differences exist. Medical men, as might be expected, are responsible for most of them, and general practitioners are perhaps as numerous in the field as obstetrical specialists. Nurses of several types have also frequently ventured into print; occasionally an author airs her views whose experience is more personal than expert. Some of these books have passed through scores of editions, and been modified by successive editors out of all resemblance to their originals. Others are brand-new from the press, and two or three of such newcomers have been reviewed in these columns within the past year or two.

To classify these books or to arrange them in an order of comparative merit and usefulness would be a heavy task, and not, probably, a very profitable one; for many are admirably adapted to their purpose, and can confidently be placed in the hands of those in need of guidance.

### The Public's Interest in Medicine.

It is indubitable that the educated public already takes a most intelligent interest in the scientific evolution of modern medicine and surgery. As long as the latter were purely empirical, governed by laws wholly mysterious to the bulk even of their professional exponents, the laity could hardly be expected or encouraged to comprehend their intricacies. Now, however, the researches of the past half-century have resulted in much more orderly and rational conceptions of the prevention and cure of disease; and those conceptions can, in outline at any rate, be made intelligible to the ordinarily clever educated man or woman. It may, therefore, be fairly supposed that the time has come to take the public a step further into confidence. With regard to surgery, and to some other branches of medical science, this has already been done by several authors, who have written chiefly for leisured and cultivated readers. Obstetric medicine stands on a rather different footing, since the developments it has undergone in the last sixty years, though equally wonderful, hardly appeal to quite the same intellectual sections of the community. The experiment of offering for lay digestion a discussion of obstetric problems, along similar lines to those already familiar in the case of, let us say, cancer or consumption, is, in these circumstances, a bold one, perhaps almost too bold, for the reason that the general level of education and of intellect amongst those whose attention is sought is insufficiently high.

### Mr. Bingham's Experiment.

However, the experiment has been made, and it will be instructive to observe, as time goes on, how it turns out. Mr. S. Bingham has written a book * for the instruction of the mother-expectant which is quite unique among this sort of literature. He deals with the signs and symptoms of pregnancy, and with the general hygiene of that state, very much in the same way as a score of such books do. This part of his work is along familiar lines; there is necessarily little that is novel about it, but the advice and instruction offered seem to be sound and good. On page 66, however, he strikes out a new line by commencing a historical survey of "child-bed fever" and its ravages among maternity wards before the days of O. W. Holmes, Semmelweis, Lister, and their successors. More than forty pages are devoted to the elaboration of this topic, which includes a great deal upon surgical cleanliness and upon puerperal fever. This section should be of the most intense interest to any highly educated woman who expects to be a mother, but we have some fear lest it should prove an alarming chapter of horrors to all but the best balanced and most scientifically minded.

The author then returns to the accustomed style in sections dealing with the engagement of nurse and doctor, after which he quotes some of the rules of the Central Midwives Board; the inclusion of these is, in our judgment, a mistake, for they do not help anyone so far as can be seen, and the formidable list of instruments, etc., might well appal any reader. Sections upon the common disorders of pregnancy follow; most of these are good, but some are likely to be above the heads of most patients and to frighten them unnecessarily.

In the following chapters, dealing with the confinement itself, an attempt is made to explain the principles of antiseptic (or aseptic) midwifery, and so to enable women to understand the why and the wherefore of the complicated ritual that goes on around their bedsides. If this information could make them expert critics of their doctors, midwives, and nurses, it would achieve a most valuable object in the prevention of slackness; but it is difficult to see how it can be possible to do this, and it is, we think, much more likely to render them apprehensive, suspicious, and generally neurotic. With a full appreciation of Mr. Bingham's motives, which are excellent, for they are the outcome of a desire to spread abroad the doctrines of cleanliness, we doubt whether the time is yet ripe for a book such as he has written. Still, he has broken new ground in a courageous way, and if events prove that his teachings are really useful to those whom he addresses, it will be an excellent thing for all concerned. We merely question the capacity of ordinary folk to receive it.

* *Words to Wives on Pregnancy and Parturition.* By S. Bingham, M.R.C.S. Pp. 201. G. Allen and Co. 1912. 3s. 6d.

